# Systems Pharmacology Dissection of the Anti-Inflammatory Mechanism for the Medicinal Herb *Folium Eriobotryae*

**DOI:** 10.3390/ijms16022913

**Published:** 2015-01-28

**Authors:** Jingxiao Zhang, Yan Li, Su-Shing Chen, Lilei Zhang, Jinghui Wang, Yinfeng Yang, Shuwei Zhang, Yanqiu Pan, Yonghua Wang, Ling Yang

**Affiliations:** 1 Key laboratory of Industrial Ecology and Environmental Engineering (MOE), Faculty of Chemical, Environmental and Biological Science and Technology, Dalian University of Technology, Dalian 116024, China; E-Mails: d11107022@mail.dlut.edu.cn (J.Z.); jhwang_dlut@163.com (J.W.); yinfengyang@yeah.net (Y.Y.); zswei@dlut.edu.cn (S.Z.); 2 System Biology Lab, University of Florida, Gainesville, FL 32608, USA; E-Mail: suchen@cise.ufl.edu; 3 Department of Electrical and Computer Engineering, University of Florida, Gainesville, FL 32608, USA; 4 School of Chemistry and Environmental Engineering, Hubei University for Nationalities, Enshi 445000, China; E-Mail: zhanglilei@outlook.com; 5 Center of Bioinformatics, College of Life Science, Northwest A&F University, Yangling 712100, China; 6 Lab of Pharmaceutical Resource Discovery, Dalian Institute of Chemical Physics, Chinese Academy of Sciences, Dalian 116023, China; E-Mail: yling@dicp.ac.cn

**Keywords:** systems pharmacology, herbal medicine, anti-inflammation, *Folium Eriobotryae*

## Abstract

Inflammation is a hallmark of many diseases like diabetes, cancers, atherosclerosis and arthritis. Thus, lots of concerns have been raised toward developing novel anti-inflammatory agents. Many alternative herbal medicines possess excellent anti-inflammatory properties, yet their precise mechanisms of action are yet to be elucidated. Here, a novel systems pharmacology approach based on a large number of chemical, biological and pharmacological data was developed and exemplified by a probe herb* Folium Eriobotryae*, a widely used clinical anti-inflammatory botanic drug. The results show that 11 ingredients of this herb with favorable pharmacokinetic properties are predicted as active compounds for anti-inflammatory treatment. In addition, via systematic network analyses, their targets are identified to be 43 inflammation-associated proteins including especially COX2, ALOX5, PPARG, TNF and RELA that are mainly involved in the mitogen-activated protein kinase (MAPK) signaling pathway, the rheumatoid arthritis pathway and NF-κB signaling pathway. All these demonstrate that the integrated systems pharmacology method provides not only an effective tool to illustrate the anti-inflammatory mechanisms of herbs, but also a new systems-based approach for drug discovery from, but not limited to, herbs, especially when combined with further experimental validations.

## 1. Introduction

Inflammation is a fundamental protective response that enables human survival when encountering a microbial invasion or injury, and also maintains the tissue homeostasis under various deleterious surroundings. It must be precisely fine-tuned and regulated, since insufficient or superfluous responses of inflammation may coequally lead to the morbidity as well as the shortening of lifespan [1]. Specifically, the deficiency of inflammatory responses always results in immunodeficiency phenomena such as cancer and infection, while the superfluity of inflammatory responses often contributes to higher incidence rate and mortality in several diseases including diabetes, Alzheimer’s disease, atherosclerosis, rheumatoid arthritis, Crohn’s disease, cerebral ischemia, multiple sclerosis, myocardial ischemia, *etc.* [1]. All these make it necessary to develop effective and safe anti-inflammatory medications.

Actually, some effective medications such as the non-steroidal anti-inflammatory agents (NSAIDs) have been developed for treating various forms of inflammatory diseases. For example, as one major group of the most widely prescribed drugs in the world, NSAIDs, like aspirin and ibuprofen, are powerful anti-inflammatory medications by virtue of their abilities to suppress the biosynthesis of prostaglandins at the level of the cyclooxygenase (COX) enzymes [2]. However, though having been identified effective for treatment of various inflammations for several decades, NSAIDs have their inherent defects,* i.e.*, severe side effects, notably the gastrointestinal hemorrhage [3], mainly due to their nonselective inhibition on two cyclooxygenase enzyme systems. In order to overcome these problems, recently, some novel selective COX2 (one isoform of COXs) inhibitors that may reduce the risk of severe gastrointestinal complications have drawn a lot of attention [4]. However, their applications are still greatly limited by the findings that selective COX2 inhibitors may also lead to a series of severe problems including hypertension, thrombosis [5], myocardial infarction and stroke [6]. Thus, developing more potent and safe anti-inflammatory drugs is still a pressing task.

Alternative herbal medicines, characterized as multiple compounds and targets, might have some advantages in developing anti-inflammatory agents in two ways: (1) being natural and therefore maybe less harmful [7] and (2) containing multiple bioactive ingredients which thus may provide synergistic and/or additive pharmacodynamic effects. Fortunately, plenty of botanic medicines like *Andrographis paniculata* (Burm. f.) Nees (Acanthaceae),* Lonicera japonica* Thunb. (Caprifoliaceae) and* Eriobotrya japonica* (Thunb.) Lindl. (Rosaceae) have been demonstrated with potent anti-inflammatory properties. For example, *Andrographis paniculata* (Burm. f.) Nees has already been used as an anti-inflammatory drug for treating rheumatoid arthritis and laryngitis for several centuries [8,9], and its major constituent andrographolide exhibits anti-inflammatory effect in LPS-stimulated RAW264.7 macrophages through the suppression of STAT3-mediated inhibition of the NF-κB pathway [10]. Likewise, *Lonicera japonica* Thunb. is also one of the most commonly used herbs in China for treating inflammation and infectious diseases for thousands of years [11,12]. Additionally, luteolin, the bioactive ingredient of *Lonicera japonica* Thunb., can block the MAPK and NF-κB signaling pathways to inhibit the inflammatory mediators release, and then exhibits an anti-inflammatory effect [13,14]. Nonetheless, due to the inherent features of herbs—*i.e.*, the complex constituents, targets and mechanisms—the exact action mechanisms of most medicinal herbs still remain unclear.

Among those botanical drugs with effective anti-inflammatory properties, *Eriobotrya japonica* (Thunb.) Lindl. which has rich resources in China and Japan, attracts our special attention. Its dried leaves, named as *Folium Eriobotryae*, have been commonly used not only for clearing away the lung-heat of antitussive and anti-inflammatory agent, eliminating phlegm and relieving coughs in China [14], but also for the treatment of stomachache, ulcers, chronic bronchitis, cancer and diabetes mellitus as Japanese folk medicine [15]. Additionally, some effective anti-inflammatory ingredients were also identified from this herb like triterpenes, flavonoids, sesquiterpenes and megastigmane glycosides [16,17]. For example, triterpene ursolic acid of *Folium Eriobotryae* exhibits significant anti-inflammatory potency by inhibiting LPS-induced IL-8 production, iNOS mRNA expression and NF-κB activation [18]; the flavonoid ingredient kaempferol also exhibits anti-inflammatory efficacy by inhibition of iNOS protein and mRNA expression, as well as of NO production and NF-κB activation [19]. Therefore, in the present work, *Folium Eriobotryae* was selected as a typical herbal medicine to probe the anti-inflammatory mechanism of herbs.

As a matter of fact, herbal medicines normally contain huge numbers of active pharmacological ingredients. Besides, herbal medicines also target several diverse genes or proteins to exert therapeutic effects, which makes the investigation of their pharmacological molecular mechanisms extremely difficult [20]. As an emerging field, systems pharmacology may provide a new approach to solve complex pharmacological problems [21]. In the context of systems biology, systems pharmacology utilizes an integrated method to investigate the drug actions ranging from molecular and cellular levels to tissue and organism levels [22]. Recently, as an important tool of systems pharmacology, network approach has proven to be useful for integrating abundant high-dimensional biological data [23], as well as for deciphering the relationships among drugs, targets, diseases and pathways [22,23,24]. Thus, it is prospected that the topological properties of the biological network may greatly enrich our knowledge about the therapeutic mechanisms underlying the multiple actions of herbs.

Here, a novel systems pharmacology method was proposed with *Folium Eriobotryae* employed as an example to dissect the underlying anti-inflammatory mechanism of herbal medicines. In detail, first of all, the compound identification program was performed to seek the active ingredients from *Folium Eriobotryae*. Secondly, target identification and validation processes were implemented to verify corresponding biological targets of these active compounds. Finally, network-based analyses among compounds, targets, pathways and diseases were carried out to explore the pharmacological mechanism of *Folium Eriobotryae* in a systematic level as well as to unfold the main targeting proteins and signaling pathways in the process of inflammatory treatment.

## 2. Results and Discussion

Owing to the complex components and synergistic nature of herbal medicines, using herb as therapeutic agent may serve as valuable resources in (a) extracting bioactive compounds for direct use as drugs; (b) discovering combination drugs or multi-target drugs; as well as (c) producing bioactive compounds of novel or known structures as lead compounds to produce entities with high activities and/or lower toxicities. Therefore, in the present work, we employed the systems pharmacology method to dissect the multiple mechanisms of action of herb *Folium Eriobotryae*, in hope that it may also provide useful information for finding novel anti-inflammatory leading compounds.

### 2.1. Active Compounds Identification

**Figure 1:**
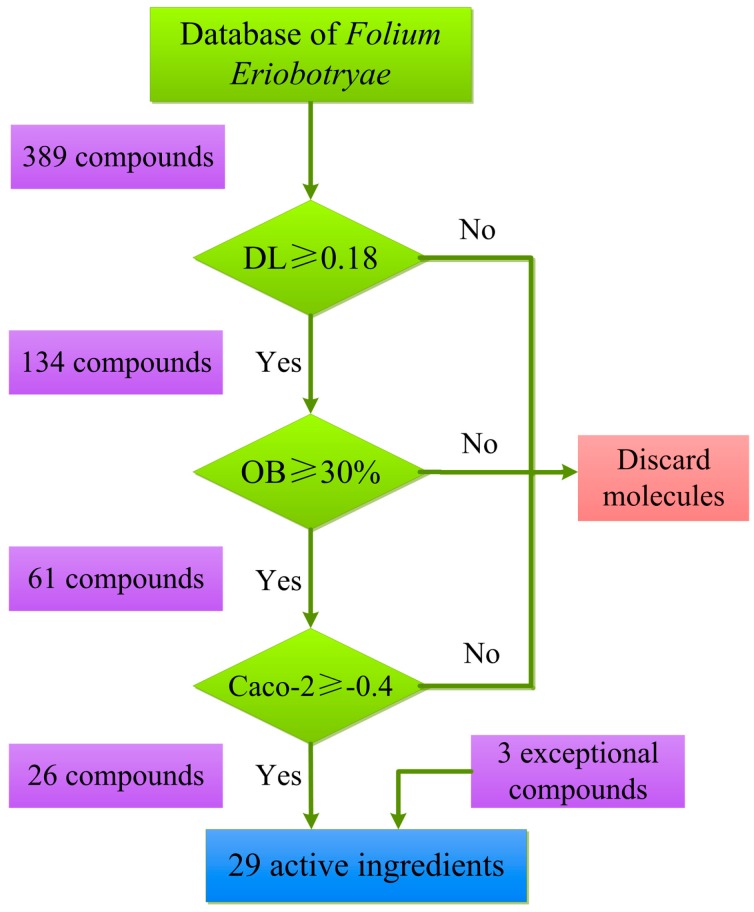
Active ingredients’ screening scheme.

A total of 389 compounds of *Folium Eriobotryae* are finally extracted from plenty literatures and TCMSP database, including 374 constituents and 15 aglycones by hydrolysis of 53 glucoside-type ingredients as displayed in Table S1. Basically, an orally administered drug must first overcome different* in vivo* barriers like the digestive tract, when it can further be transported into the blood circulation to arrive at the specific tissues or organs, and then exerts pharmacological effects [25]. Therefore, in the present work, in order to filter the potential bioactive compounds from *Folium Eriobotryae*, three crucial ADME parameters (drug-likeness, oral bioavailability and Caco-2 permeability) as typical indicators were chosen to evaluate the abilities of herbal ingredients to overcome these barriers, with the detailed screening process displayed in Figure 1.

#### 2.1.1. Drug-likeness Evaluation

As a matter of fact, druggable compounds usually have some commonalities, such as functional groups and physical properties [26]. Therefore, assuring molecules with a high drug-likeness (DL) index can increase the possibility of therapeutic success, and the higher DL value a molecule has, the larger possibility there is that it may possess certain biological properties. Here, an optimal DL model based on 6511 molecules in the DrugBank database were constructed to prescreen potential active ingredients. Among the 389 compounds, 134 ingredients pass through the threshold of DL ≥ 0.18; thus, they may exhibit favorable drug-likeness features. Actually, some of them have been experimentally validated with various biological activities. For examples, the DL value of oleanolic acid is high (0.76), indicating its large possibility to become a potential drug, which corresponds well with the fact that oleanolic acid has already achieved satisfactory effect in clinical trials in 1977 ever since its successful use as a hepatoprotective drug to treat liver disease in China [27,28]. Furthermore, this compound also exhibits anti-inflammatory and anti-hyperlipidemic properties in several animal models [27,29,30]. Moreover, maslinic acid with DL value of 0.74 possesses anti-inflammatory effect against carrageenan-induced edema and inhibitory effect on histamine-induced ileum contraction [31]; As to 2α-hydroxy ursolic acid (DL = 0.74), it shows inhibitory effects against series of cell lines including HL-60, BGC, Bel-7402 and Hela ones [32]. 

#### 2.1.2. Oral Bioavailability Prediction

Compounds possessing satisfactory DL indexes have high probabilities to become promising candidate drugs, but this is not absolutely certain for the reason that the DL index is a unified descriptor. To ensure the reliability of screening model, some major specific descriptors of molecular properties including oral bioavailability (OB) and Caco-2 permeability should also be employed as screening parameters [33]. Among these two profiles, OB value is governed by the dissolution of drug in the gastrointestinal tract, the intestinal and hepatic first-pass metabolism, as well as the intestinal membrane permeation, which makes it one of the major pharmacokinetic profiles in drug development. Therefore, presently, the OB model is further utilized to prescreen active ingredients from medicinal herb. As a result, 61 out of the above DL-screened 134 constituents (about 46%) exhibit proper oral bioavailabilities (OB ≥ 30%). As a matter of fact, many compounds with high OB values, such as chlorogenic acid (OB = 63%) and pomolic acid (OB = 75.45%), have already been identified as bioactive ingredients. For examples, chlorogenic acid with dose of 50 mg/kg inhibits carrageenin-induced paw edema in the early phase of rat model, and with the same dose this compound also exhibits analgesic activities in the late phase of formalin-induced pain test [34]. Additionally, pomolic acid shows anti-inflammatory activity through inhibiting the carrageenan-induced paw oedema by 37% (1 h) at a dose of 125 mg/kg in mouse model [35]. Thus, these 61 compounds were finally extracted for further analysis.

#### 2.1.3. Caco-2 Permeability Screening

Besides oral bioavailability, Caco-2 permeability is another widely used parameter, which is employed to evaluate the absorption rate of candidate compound across the intestinal epithelial barrier. Therefore, in the present work, Caco-2 permeability model is introduced to evaluate the absorption rates of above 61 molecules. As a consequence of this screening, 26 compounds (with both high OB and DL values) are found meeting the threshold requirement of Caco-2 permeability (≥−0.4). Corresponding to this, several ingredients possessing higher Caco-2 permeability values have also been confirmed with biological activities, like β-sitosterol (Caco-2 = 1.33), methyl ursolate (0.92) and oleanolic acid (0.61). For instances, β-sitosterol exerts anti-inflammatory effect through suppressing oedema formation by 52% with dose of 0.5 mg/ear in mice model [36], while an* in vitro* study [37] suggests that the anti-inflammatory activity of β-sitosterol is involved in arachidonic acid cascade. In addition, methyl ursolate exhibits the strong inhibitory effect (50% inhibitory dose, ID_50_ = 0.43 mg/ear) on TPA-induced inflammation in mice model, and also displays inhibitory effect with IC_50_ value of 477 mol ratio/32 p mol TPA [38].

#### 2.1.4. Exceptional Molecules

In fact, reality constraints of most biological models force the dependent variables to lie in some finite bounds [39], which may cause models to have some limitations, and our models are no exception. Because not only the chemo-physical properties of herbal ingredients should be weighted when building a model to explore the critical factors influencing the therapeutic effects, but also other characteristics of a herbal medicine like the ingredient contents should also be taken into consideration, due to the fact that under certain circumstances, they may also impact the pharmaceutical effects of the herb. Thus, in order to avoid possible omissions of active ingredients during our prescreening process, a large-scale text mining was also performed to acquire all existing candidate compounds of *Folium Eriobotryae*, which ends up with three exceptional molecules when compared with our screened results as mentioned above.

The first one is ursolic acid which has been experimentally validated to be bioactive ingredient of *Folium Eriobotryae* exhibiting anti-inflammatory and anti-hyperlipidemic properties in animal models [27,32]. However, it only obtains a low OB predicted value (about 10%), which is not surprising due to its poor solubility [40]. Factually, the average content of ursolic acid reaches 7.57 mg/g in the herb [41] which may greatly increase its absolute OB value, and in this way compensate for and assure the acid’s pharmaceutical effects.

The other two compounds are chlorogenic acid and rutin. Chlorogenic acid has anti-inflammatory effect through attenuating the activation of JNK/AP-1 and NF-κB signaling pathways [34,42,43]. However, its Caco-2 permeability is very poor (−1.07). The reason may be on account of the fact that this molecule can be further hydrolyzed into caffeic acid by esterase hydrolysis of the colonic microflora [44]. Whereas, the latter can be efficiently absorbed (OB = 51.08% and Caco-2 = 0.24). Factually, caffeic acid has been identified as having antioxidant properties to scavenge ABTS^•+^, DPPH^•^ and superoxide anion radical, as well as to reduce power and metal chelating [45]. Additionally, it also exhibits anti-inflammatory property in cardiac tissue of diabetic mice [46]. Thus, caffeic acid as a metabolite should also be considered as an active ingredient, despite its low DL index (0.05) less than 0.18. Similarly, rutin has very low Caco-2 permeability as −1.91, but its metabolite quercetin (Caco-2 = 0.02) can easily pass through the colon barrier to exert biological effects [47], which we assume is the reason why rutin still possesses high biological activities in* in vivo* models [39].

Thus, in the end, the compounds chlorogenic acid, caffeic acid and rutin are also added into the active ingredients database. By combining the modeling results, finally, 29 compounds are selected as the potential active ingredients of *Folium Eriobotryae*, with their ADME parameters and biological information displayed in Table 1. Out of these chemicals, nearly two thirds (19 compounds) have been experimentally validated with various bioactivities, such as anti- inflammatory, oxidant, diabetic, cancer effects. Interestingly, among these 19 active ingredients, 15 compounds which are mostly classified as triterpene acids and flavonoids exhibit anti-inflammatory activities as shown in Table 1. All these results well demonstrate the feasibility and effectiveness of our prescreening models.

**Table 1:** The pharmacokinetic and biological information of potential active ingredients of *Folium Eriobotryae.*

No.	Ingredient	Structure	OB (%)	Caco-2	DL	Bioactivity (PMID)
M_01	β-sitosterol	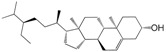	45.04	1.33	0.75	anti-inflammation (6967611); antipyretic (6967611); anthelminthic & anti-mutagenic activities (12203259); anti-oxidative (16508147)
M_02	tormentic acid	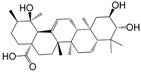	33.73	−0.20	0.71	anti-inflammation (12007601); anti-HIV (12907261); antidiabetic (11830140); antitumor-promoting (14745168)
M_03	sanguisorba,officinalis-7	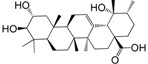	35.32	−0.25	0.71	
M_04	rutin	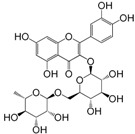	35.45	−1.91	0.28	anti-inflammation (11680812); antioxidant (10904145)
M_05	quercetin	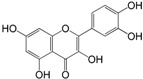	31.36	0.02	0.28	antioxidant (9414116); anti-inflammation (18549926); pro-apoptotic (14688022)
M_06	pomolic acid	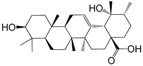	75.45	0.26	0.73	anti-inflammation (18260049); anticancer (22223345); anti-apoptotic (15694664); anti-HIV (9748372)
M_07	oleanolic acid	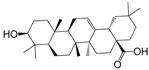	66.91	0.61	0.76	anti-inflammation (1359067); antioxidant (22891614); anti-HIV (9748372); antidiabetic (18066109); hepatoprotection (18066109); antibacterial (18069238)
M_08	methyl ursolate	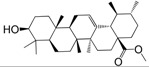	47.63	0.92	0.74	anti-inflammation (16204964)
M_09	methyl maslinate	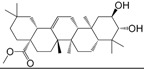	42.79	0.32	0.72	
M_10	methyl betulinate	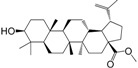	43.41	0.85	0.76	
M_11	methyl arjunolate	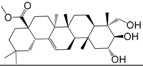	56.25	−0.05	0.70	anti-inflammation (16204964)
M_12	maslinic acid	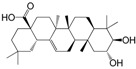	55.84	0.04	0.74	anti-inflammation (3769076); anti-HIV (8759159); antioxidant (12802735); anti-tumor (20509140); antiangiogenic (21175131)
M_13	linguersinol	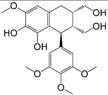	48.60	−0.17	0.54	
M_14	kaempferol	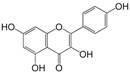	65.70	0.27	0.24	anti-inflammation (17666881); analgesic (17666881); antioxidant (17551714); anti-bacteria (15234754)
M_15	delta 7-stigmastenol	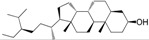	40.40	1.30	0.75	
M_16	2α-hydroxy ursolic acid	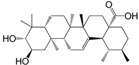	39.30	0.03	0.74	anti-cancer (15922841)
M_17	arjunolic acid	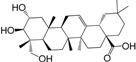	43.27	−0.27	0.72	antioxidant (18273903)
M_18	3-*O*-*trans*-*p*-coumaroyltormentic acid	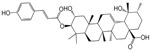	46.94	−0.17	0.34	anti-inflammation (16204964)
M_19	3-*O*-*trans*-caffeoyltormentic acid	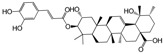	36.33	−0.07	0.32	
M_20	3-*O*-*cis*-*p*-coumaroyltormentic acid	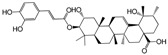	33.35	−0.19	0.34	anti-inflammation (16204964)
M_21	3-epiursolic acid	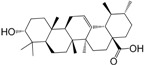	39.92	0.51	0.75	
M_22	2α,3α-dihydroxyursolic acid	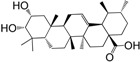	37.73	0.25	0.74	
M_23	2α,3α-dihydroxyurs-12-en-28-oic acid	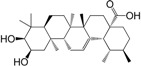	45.16	0.21	0.74	
M_24	2α,3α,23-trihydroxyolean-12-en-28-oic acid	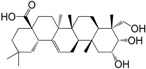	41.34	−0.27	0.72	anti-inflammation (16204964)
M_25	2α,3α,19α-trihydroxy-12-oleanen-28-oic acid	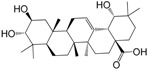	30.03	−0.18	0.72	
M_26	2α,19α-dihydroxy-3-oxo-urs-12-en-28-oic acid	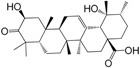	46.95	−0.30	0.71	anti-HIV (12907261)
M_27	ursolic acid	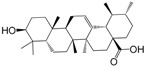	9.95	0.79	0.66	anti-inflammation (19051345); anti-HIV (8759159); hepatoprotection (8847885); antiangiogenic (21175131); anti-cancer (15922841); antibacterial (18069238)
M_28	chlorogenic acid	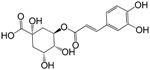	63.12	−1.07	0.33	antioxidant (12771329); anticarcinogenic (12771329); anti-inflammation (17077520); analgesic (17077520)
M_29	caffeic acid	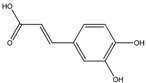	51.08	0.24	0.05	antioxidant (16243424); anticoagulant (19678956); anti-inflammation (19678956)

### 2.2. Target Identification

Indisputably, herbal medicines exert their therapeutic effects through the synergistic effects of multiple compounds and targets. Thus, besides exploring active ingredients, the therapeutic targets exploration is also a necessity. In the present work, for comparison, first of all, we screened out all therapeutic proteins being targeted by FDA-approved anti-inflammatory agents from DrugBank and TTD databases. The results of target exploration show that, among the 29 active ingredients obtained as described above, 25 compounds are found interacting with 232 targets, while the other four molecules have no targets. Since our work attempts to elucidate the anti-inflammatory effect of *Folium Eriobotryae*, these target proteins were further sent to TTD and PharmGKB databases to identify if they have relationships with inflammatory diseases. As a result, 53 inflammation-related targets which have interactions with 13 corresponding compounds were screened out. After docking validation process, 43 targets linked with 11 active ingredients are finally considered as the candidate anti-inflammation-related targets and compounds of this herb. The detailed information of the 43 target proteins is described in Table 2.

**Table 2:** The information of anti-inflammation-related targets of *Folium Eriobotryae.*

No.	Protein Name	Gene Name	UniProt ID
P_01	3-hydroxy-3-methylglutaryl-Coenzyme A reductase	HMGCR	P04035
P_02	Aldose reductase	AKR1B1	P15121
P_03	Arachidonate 15-lipoxygenase	ALOX15	P16050
**P_04**	**Arachidonate 5-lipoxygenase**	**ALOX5**	**P09917 **
P_05	Baculoviral IAP repeat-containing protein 5	BIRC5	O15392
P_06	C-C motif chemokine 2	CCL2	P13500
P_07	CD40 ligand	CD40LG	P29965
P_08	Cell division protein kinase 2	CDK2	P24941
P_09	Cholinesterase	BCHE	P06276
P_10	C-X-C motif chemokine 10	CXCL10	P02778
P_11	C-X-C motif chemokine 2	CXCL2	P19875
P_12	Cytochrome P450 1A2	CYP1A2	P05177
P_13	Epidermal growth factor receptor	EGFR	P00533
P_14	E-selectin	SELE	P16581
P_15	Glycogen synthase kinase-3 beta	GSK3B	P49841
P_16	Heme oxygenase 1	HMOX1	P09601
P_17	Inhibitor of nuclear factor kappa-B kinase subunit alpha	CHUK	O15111
P_18	Inhibitor of nuclear factor kappa-B kinase subunit beta	IKBKB	O14920
**P_19**	**Intercellular adhesion molecule 1**	**ICAM1**	**P05362**
P_20	Interleukin-1 alpha	IL1A	P01583
P_21	Interleukin-1 beta	IL1B	P01584
P_22	Interleukin-10	IL10	P22301
P_23	Interleukin-2	IL2	P60568
P_24	Interleukin-6	IL6	P05231
P_25	Interleukin-8	IL8	P10145
P_26	Mitogen-activated protein kinase 14	MAPK14	Q16539
P_27	Mitogen-activated protein kinase 8	MAPK8	P45983
P_28	Muscarinic acetylcholine receptor M1	CHRM1	P11229
P_29	NF-kappa-B inhibitor alpha	NFKBIA	P25963
P_30	Nitric-oxide synthase, endothelial	NOS3	P29474
P_31	Ornithine decarboxylase	ODC1	P11926
P_32	Osteopontin	SPP1	P10451
P_33	Peroxisome proliferator-activated receptor alpha	PPARA	Q07869
**P_34**	**Peroxisome proliferator-activated receptor delta**	**PPARD**	**Q03181**
**P_35**	**Peroxisome proliferator-activated receptor gamma **	**PPARG**	**P37231**
P_36	Phospholipase A2	PLA2G1B	Q9BS22
P_37	Poly [ADP-ribose] polymerase 1	PARP1	P09874
**P_38**	**Prostaglandin G/H synthase 1**	**COX1**	**P23219**
**P_39**	**Prostaglandin G/H synthase 2 **	**COX2**	**P35354**
P_40	Signal transducer and activator of transcription 1-alpha/beta	STAT1	P42224
P_41	Transcription factor AP-1	JUN	P05412
P_42	Transcription factor p65	RELA	Q96F54
P_43	Tumor necrosis factor	TNF	P01375

Targets that shared by the herbal ingredients and FDA-approved drugs are marked in bold.

By analyzing all targets in Table 2, two interesting phenomena were observed. On one hand, seven active herbal ingredients target six therapeutic targets of FDA-approved anti-inflammatory agents*—i.e.*, COX2, COX1, ALOX5, ICAM1, PPARG and PPARD—indicating that *Folium Eriobotryae* may have similar pharmacological mechanisms to Western medicines. On the other hand, two categories of inflammation closely-related proteins are found among these targets,* i.e.*, pro-inflammatory cytokines (such as TNF, IL1A, IL1B, IL2, IL6 and IL8) and anti-inflammatory cytokines (like IL4 and IL10), which indicates that *Folium Eriobotryae* may also regulate these two categories of targets to exert synergistic or additive anti-inflammatory effect. In summary, compared to the usual mono-therapy for patients with Western drugs, the herb *Folium Eriobotryae* may have advantages for treatment of inflammation diseases in both regulating multiple inflammatory pathways and exerting additive or synergistic effects for improving the outcome. 

### 2.3. Network Construction and Analysis

In the field of systems pharmacology, network analyses provide efficient tool for enhancing the understanding of action mechanisms of multi-drugs in the context of regulatory networks [22], and it is also useful for organizing high-dimensional biological data and extracting useful information. In view of the fact that herbal medicine exerts pharmacological effects through multiple compounds, targets and mechanisms, using the biological network to dissect action mechanisms of herbal medicines is a necessity [48]. Factually, the biological network has already been successfully used in deciphering the molecular mechanism of several traditional Chinese medicines which are used in treating stroke [49] and depression [50].

In this work, to better understand the therapeutic mechanism of *Folium Eriobotryae*, four biological networks were constructed, including D–T, I–T, T–P and T–D networks. Firstly, for comparison, the anti-inflammatory drug-target network (D–T network) for western medicines was constructed based on all FDA-approved anti-inflammatory therapeutic drugs and targets from DrugBank and TTD databases. Secondly, in order to better decipher the anti-inflammatory mechanism of *Folium Eriobotryae*, the anti-inflammatory ingredient-target network (I–T network) of this herb was built by employing all anti-inflammation-related active ingredients and their corresponding targets. Subsequently, in order to elucidate the major signaling pathways implicated in inflammatory treatment by *Folium Eriobotryae*, 16 inflammation-related biological pathways were extracted to construct the target–pathway network (T–P network) with corresponding targets of herbal compounds. Finally, target-disease network (T–D network) was achieved by connecting all active herbal ingredients to their related diseases revealing the potential therapeutic effects of *Folium Eriobotryae*.

#### 2.3.1. Anti-Inflammatory D-T Network for Western Medicines

**Figure 2:**
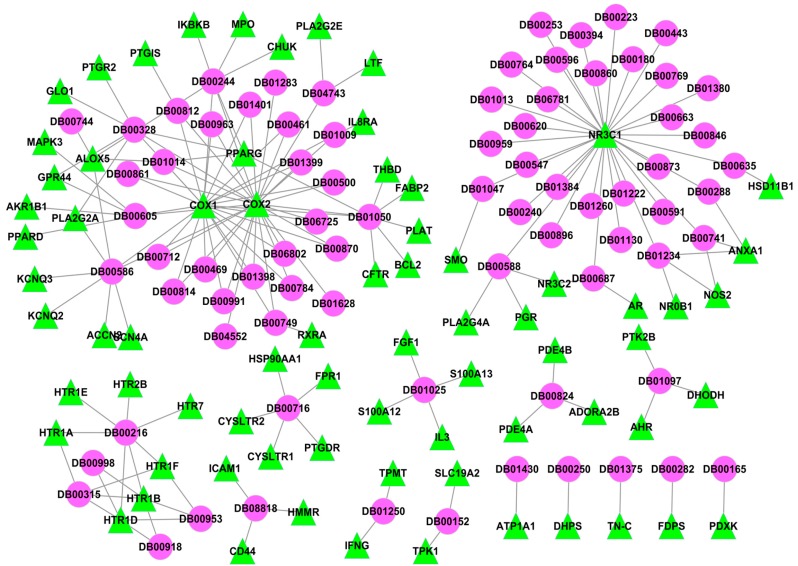
The D–T network where fuchsia circular nodes represent the DrugBank ID of anti-inflammatory drugs and lime diamond nodes represent the therapeutic targets, respectively.

To compare the therapy mechanism differences between herbs and conventional FDA-approved drugs in treating inflammatory diseases, we extracted all therapeutic targets of anti-inflammatory drugs from databases of the DrugBank and TTD, and then built a drug–target network of Western medicine. In the present work, until 30 December 2013, a total of 78 FDA-approved anti-inflammatory drugs and their corresponding 72 target proteins had been extracted, which then were employed to generate the bipartite graph of D-T network as shown in Figure 2.

As displayed in Figure 2, the D–T network embodies 150 nodes (78 drugs and 72 therapeutic targets) and 176 edges. In this network, three targets,* i.e.*, glucocorticoid receptor (NR3C1), prostaglandin G/H synthase 2 (COX2) and prostaglandin G/H synthase 1 (COX1), which are linked with 32, 28 and 24 drugs, respectively, become the major therapeutic targets for the anti-inflammatory drugs due to their dominant positions in this network. Taking NR3C1 as an example, a kind of widely used anti-inflammatory drug, the steroids like prednisone (DB00635 as displayed in Figure 2), betamethasone (DB00443) and methylprednisolone (DB00959) exhibit anti-inflammatory properties by activating NR3C1 which regulates the transcription of some response genes containing the expression inducible COX2 and NOS2 [51]. Additionally, cyclooxygenases (COX1 and COX2) are targeted by another widely used type of anti-inflammatory agent, NSAIDs, like ibuprofen (DB01050 as shown in Figure 2), meloxicam (DB00814) and indomethacin (DB00328). Additionally, NSAIDs exhibit their anti-inflammatory effects by inhibiting cyclooxygenase activity which can diminish the prostaglandin production [52]. Therefore, we conclude that these three major therapeutic targets (NR3C1, COX1 and COX2) are closely related to the anti-inflammatory functions of Western medicine.

#### 2.3.2. Anti-Inflammatory I-T Network for *Folium Eriobotryae*

**Figure 3:**
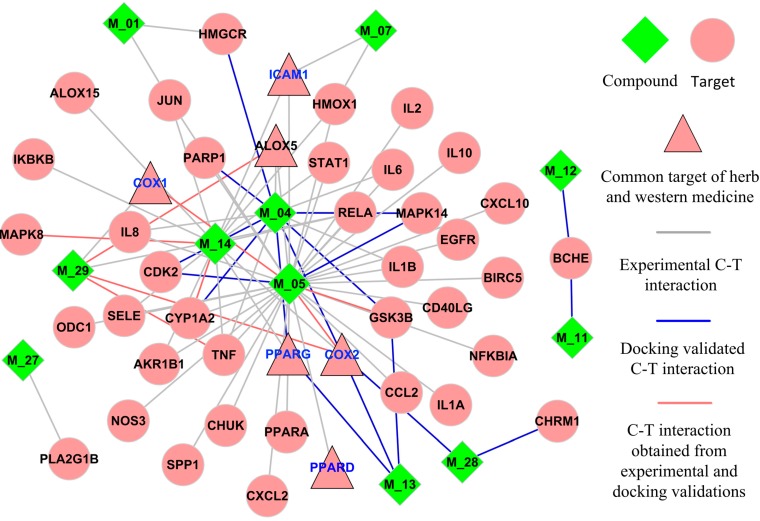
I-T network for *Folium Eriobotryae*.

To decipher the action mechanism of *Folium Eriobotryae* against the inflammatory diseases, its I-T network was constructed based on all active compounds and their inflammation-related targets (Figure 3). As shown in Figure 3, this network is composed of 54 nodes (11 active ingredients and 43 targets) and 82 interactions.

From this I-T network, four characteristics of *Folium Eriobotryae* are observed: (1) The promiscuous properties of its ingredients. The “promiscuous property” of a compound means that a single molecule selectively targets on multiple receptors [53]. As displayed in Figure 3, almost 50% of active compounds are connected with more than two targets, which well-reflect the promiscuous property of *Folium Eriobotryae*; (2) The existence of highly interconnected compounds. Interconnectedness in a network means the average length of the shortest paths between any two nodes [54] and nodes with high degrees are responsible for the high interconnectedness of this network [55]. Thus, in this network, some compounds with multiple targets are observed to be responsible for the high interconnectedness of the I–T network, especially for high-degree compounds M_05 (quercetin, degree = 38), M_14 (kaempferol, degree = 15), M_04 (rutin, degree = 14) and M_29 (caffeic acid, degree = 5). Their degrees are much larger than the average value (3.04), and they thus become highly interconnected compounds of this network. Taking quercetin as an example, it is not only an inhibitor of PPAR1 [56], but also a weak partial agonist of PPARG reporter gene assay [57]; (3) The presence of highly interconnected targets. Obviously, in the I–T network, the degrees of targets COX2, ALOX5, PPARG, TNF and RELA are 6, 4, 4, 4 and 4, respectively, all higher than the average degree of target nodes (1.91), which indicates their indispensable roles in the I–T network. Thus, these five highly interconnected targets (COX2, ALOX5, PPARG, TNF and RELA) should be treated as the crucial targets of *Folium Eriobotryae*; (4) Synergistic or additive therapeutic actions of herbal ingredients. As mentioned in the C–T network, COX2 is the major therapeutic targets of inflammation. Additionally, protein PPARG [58], TNF [59] and MPK14 [60] are also identified as the potential therapeutic targets of inflammatory diseases. Fortunately, for the herb *Folium Eriobotryae*, several ingredients are involved in regulating these known or potential therapeutic targets, which may provide synergistic and/or additive therapeutic effects in patients with inflammatory diseases.

Furthermore, by comparing and analyzing the D–T and I–T networks, we can observe two interesting phenomena. On one hand, six major targets*—i.e.*, COX2, ALOX5, PPARG, ICAM1, COX1 and PPARD—exist in both networks. Among them, COX2, ALOX5 and PPARG are regarded as the crucial targets of *Folium Eriobotryae* as mentioned above. Therefore, we deduce that *Folium Eriobotryae* may show similar anti-inflammatory mechanisms as Western medicines. Secondly, mutual interactions and constraints among multiple targets may counteract or reduce the side effects of *Folium Eriobotryae*. For instance, as mentioned above, NSAIDs non-selectively target on cyclooxygenase enzymes (COX1 and COX2), especially COX1, and in this way increase the risk of serious gastrointestinal adverse events. Similarly, for *Folium Eriobotryae*, its ingredients M_05 and M_29 also interact with both COX1 and COX2, which may also produce certain side effects. However, no literature about the side effects of *Folium Eriobotryae* has been reported up-to-date. Why? By analyzing the I–T network, we find that the major reason may be related to the other seven targets*—i.e.*, MAPK14, PPARG, RELA, TNF, MAPK8, HMOX1 and CXCL10—which are also implicated in gastrointestinal diseases. The interactions and constraints among these seven targets together with COX1 may offset the gastrointestinal adverse effects. Indeed, *Folium Eriobotryae* has no side effect on the gastrointestinal tract, and has been used for a long time in the treatment of gastroenteric disorders in Japan [61], which may be attributed to the contributions of these targets. Thus, we conclude that, exhibiting similar anti-inflammatory mechanisms as Western medicines, *Folium Eriobotryae*, characterized as multi-targets, may exhibit therapeutic superiority (less or no side effects) for treating inflammatory diseases in comparison to Western medicines.

#### 2.3.3. Target-Pathway Network of *Folium Eriobotryae*

**Figure 4:**
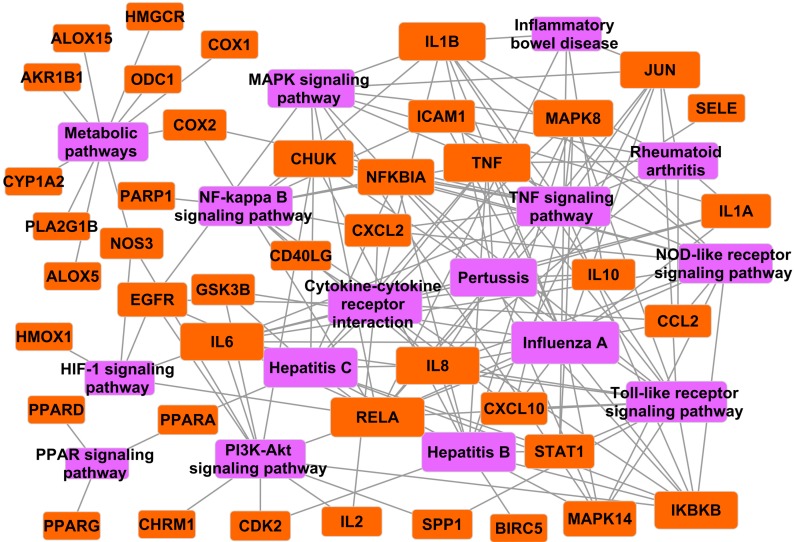
T-P network. The orange nodes represent the targets, while the purple ones the pathways. Node size is proportional to its degree.

Depending on the specific inflamed tissue or organ involved, inflammatory process generates diverse diseases. However, all of the disorders have common features or conjoint cellular processes, like the activation of a stress signaling pathway and the concomitant production of inflammatory cytokines [62]. Here, to further elucidate the action mechanism of *Folium Eriobotryae*, the T–P network was constructed in this section. Firstly, 16 inflammation-related KEGG pathways, such as MAPK signaling pathway, NF-κB signaling pathway, rheumatoid arthritis, influenza A and TNF signaling pathway were extracted from the KEGG database. Then, our screened inflammation-related targets were mapped onto the 16 pathways. The subsequent modeling results show that, only one target,* i.e.*, BCHE does not get involved in any related pathway. Thus, based on all other 42 targets and corresponding signaling pathways, a T–P network was erected as displayed in Figure 4.

Clearly, Figure 4 depicts those pathways that are intensively connected to corresponding targets. For instance, influenza A exhibits the highest number of target connections (degree = 16), as followed by the toll-like receptor signaling pathway with 14 targets, and NF-κB pathway with 12 ones, respectively. By further analyzing the T–P network, we observe an interesting phenomenon that some ingredients bind to the different targets of the network, which, however, turn out belonging to the same biological pathway, and in this way these ingredients may exert complementary actions for *Folium Eriobotryae* to exhibit synergistic anti-inflammatory effects. For example, seven ingredients including M_04, M_05, M_07 (oleanolic acid), M_13, M_14, M_28 and M_29 are involved in mediating the major components of NF-κB signaling pathway like COX2, TNF, RELA, ICAM1 and PARP1, which may contribute to the synergistic therapeutic effects for patients with inflammatory diseases. Additionally, five compounds of *Folium Eriobotryae* including M_01 (β-sitosterol), M_04, M_05, M_14 and M_29 may disturb Toll-like receptor signaling pathway through regulating its related 14 proteins like TNF, RELA and JUN, and then provide synergistic therapeutic effects to benefit patients.

In addition, by observation of the T–P network, we find that NF-κB and MAPK signaling pathways should be considered as the major anti-inflammatory pathways of *Folium Eriobotryae*, due to their topological characteristics in the net and their core roles as cross-talk pathways with the other 15 pathways. In detail, firstly, NF-κB and MAPK signaling pathways respectively hold 12 and 10 proteins, which are responsible for the high interconnectedness of the T–P network. Additionally, the crucial targets existing in the I-T network, such as COX2, PPARG, TNF and RELA, are involved in these two pathways. Furthermore, we discover that NF-κB and MAPK signaling pathways being the major cross-talk pathways play crucial roles in several other pathways. For instance, NF-κB and toll-like receptor signaling pathways are coupled together to activate the intracellular signaling cascades, and subsequently regulate IKBKB (inhibitor of nuclear factor kappa-B kinase subunit beta) and downstream protein NF-κB. Indeed, IKBKB is essential for TNF and IL1-induced NF-κB activation and therefore becomes a potential target for strategy aimed at suppressing proinflammatory gene expression for the treatment of several inflammatory diseases [63]. For instance, the anti-inflammatory effect exerted by compound M_05 occurs, at least partially, through the binding to IKBKB, which finally interferes both NF-κB and toll-like receptor signaling pathways. In conclusion, we assume that* Folium Eriobotryae* may exert anti-inflammatory effect primarily through the regulation of NF-κB and MAPK signaling pathways.

#### 2.3.4. Target-Disease Network

**Figure 5:**
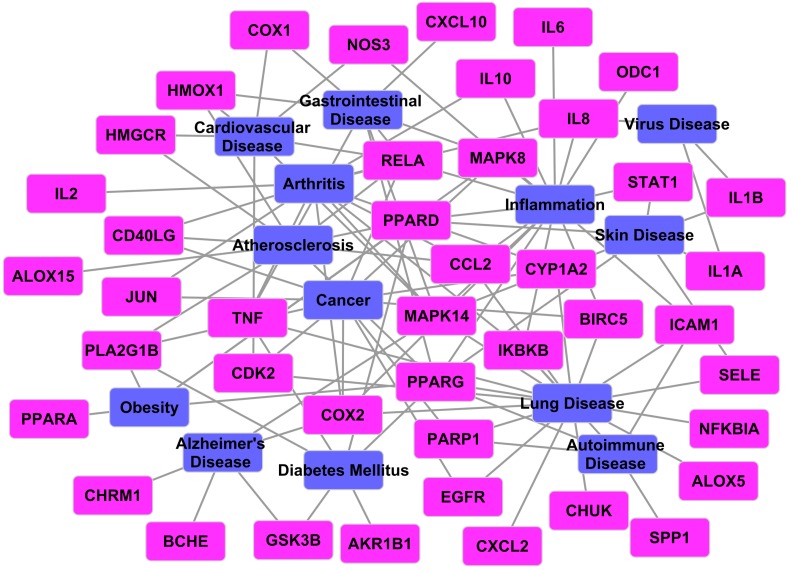
T-D network. The blue nodes show the representative diseases, while the magenta ones the targets.

According to the expanded human disease network, the majority of diseases have some common genetic origins with others. Furthermore, it is well known that inflammatory responses can result in several diseases, including infection, cancer, atherosclerosis, diabetes and rheumatoid arthritis [1]. Thus, we speculate that herb *Folium Eriobotryae* may be involved in treating other diseases besides inflammation. To prove this, here, all targets were sent to the databases of PharmGKB and TTD to find their corresponding diseases, and then a T–D network (as shown in Figure 5) was constructed based on all 43 targets and their corresponding diseases.

As displayed in Figure 5, 43 proteins are connected with 13 disorders like inflammation, lung disease, arthritis, cancer and atherosclerosis. Considering the fact that all targets in this network are implicated in inflammation, we suggest that inflammation may be closely involved in the pathogenesis of other 12 disorders. Indeed, inflammation has been proved to be implicated in many diseases. For example, Balough* et al.* [64] showed that inflammation was present early in the course of cystic fibrosis lung disease. Moreover, it had been shown that chronic inflammation was a critical component of cancer growth and metastasis, and reducing inflammation with the appropriate mode would abrogate cancer and recuperate tissue health [65,66]. Therefore, we assume that using *Folium Eriobotryae* to treat inflammation may be beneficial for the improvement of other diseases which include lung disease, arthritis, cancer, atherosclerosis and another three diseases.

Factually, over half of the diseases obtained from this T–D network are strongly supported by the results of the series of pharmacological function studies on *Folium Eriobotryae* (as displayed in Table 3). For instance, the extracts of *Folium Eriobotryae* can be used as anti-metastatic agent for cancer metastasis therapy [67,68]. Furthermore, triterpenes extracted from this herb also display anti-proliferative and apoptosis induction activities against cancer cells [69]. Likewise, the total sesquiterpenes [68], triterpene acids [70], flavonoids [70] and 70% ethanol extracts [71] of *Folium Eriobotryae* all exhibit anti-hyperglycemic effects. All these prove that this herb is a promising remedy for the treatment of diabetes mellitus and cancer. Besides, *Folium Eriobotryae* has also been used for a long time for the empirical treatment of gastrointestinal [71], skin [72] and lung diseases [73]. Therefore, we speculate that in addition to other eight pharmaceutical effects (including the anti-inflammation, cancer and diabetes, as well as the treatment of lung, skin, Alzheimer’s, gastrointestinal and virus diseases as listed in Table 3), *Folium Eriobotryae* may still have therapeutic potential for treatment of another five “not proven” diseases including cardiovascular disease, arthritis, atherosclerosis, autoimmune disease and obesity (as displayed in Table 3), which may be the subsequent research objects of pharmacological studies for this herb.

**Table 3:** The pharmacological actions of *Folium Eriobotryae.*

Actions	References (PMID)
Anti-inflammation	Banno N, *et al.* [38]; Huang Y, *et al.* [74]; Uto T, *et al.* [75]; Ge JF, *et al.* [73]; Kim SH, *et al.* [76]; Shimizu M, *et al.* [31]; Choi YG, *et al.* [77]; Cha DS, *et al.* [78]
Anti-cancer	Banno N, *et al.* [38]; Ito H, *et al.* [61]; Ito H, *et al.* [79]; Kim SH, *et al.* [80]
Treatment of cardiovascular diseases	Not proved
Treating lung diseases	Ge JF, *et al.* [73]; Lee CH, *et al.* [18]
Anti-diabetes	Li WL, *et al.* [71]; Lü H, *et al.* [70]; Chen J, *et al.* [81]; Qa'dan F, *et al.* [82]; De Tommasi N, *et al.* [83]; Chen J, *et al.* [72]; Tanaka K [84]; Noreen W, *et al.* [85]; Gumy C, *et al.* [86]; Kim SH, *et al.* [80]; Lü H, *et al.* [87]; Chen J, *et al.* [72]
Treating skin diseases	Shimizu M, *et al.* [72]; De Tommasi N, *et al.* [88]
Antirheumatic activity	Not proved
Neuroprotective effects	Kim MJ, *et al.* [89]
Treatment of gastroenteric disorders	Ito H, *et al.* [61]
Anti-atherosclerosis	Not proved
Treatment of autoimmune disease	Not proved
Antiviral effect	N De Tommasi, *et al.* [88]
Anti-obesity	Not proved

## 3. Materials and Methods

### 3.1. Database Establishment

In total, 374 ingredients of *Folium Eriobotryae* were extracted from literatures and the Traditional Chinese Medicine System Pharmacology Database and Analysis Platform (TCMSP, available online: http://tcmspnw.com), and then added into the ingredient database of *Folium Eriobotryae*. All structures of these compounds were saved as mol2 format, and then optimized by Sybyl 6.9 (Tripos Associates, St. Louis, MO, USA) with the same parameters as described in our previous work [90]. It is generally known that deglycosylation by colonic bacteria is a critical step in the absorption and metabolism of glycosides in humans. Therefore, based on the rule of glycosidase hydrolysis reaction, 53 ingredients with glycosyls were further deglycosylated, and the corresponding resulted 15 aglycones were added into ingredient database for further research. All information of the 389 ingredients is displayed in Table S1.

### 3.2. Pharmacokinetic Prediction

In drug development, poor pharmacokinetic properties of drugs become the major cause of costly late-stage failures. Thus, early ADME (absorption, distribution, metabolism and excretion) evaluation is extremely important [91]. Due to the disadvantages of biological experiments as time-consuming and high-cost, identification of ADME properties by *in silico* tools has now become an inevitable paradigm in pharmaceutical research. In this work, three ADME-related models, including the evaluation of oral bioavailability (OB), Caco-2 permeability and drug-likeness (DL), were employed to identify the potential bioactive compounds of *Folium Eriobotryae*.

***Drug-likeness evaluation.*** Drug-likeness is a qualitative profile used in drug design to evaluate whether a compound is chemically suitable for drug, and how drug-like a molecule is with respect to parameters affecting its pharmacodynamic and pharmacokinetic profiles which ultimately impact its ADME properties [26]. In our present work, we developed a robust *in silico* model to predict a compound’s DL index, which was supported by a database of 6511 structurally diverse drugs and drug-like molecules from DrugBank database. In this model, we introduced DL-related parameters as many as possible, which ends up with 1533 descriptors containing constitutional descriptors (like molecular weight), one-dimensional parameters (such as log*P*, number of H-donors and H-acceptors), two-dimensional factors (for example, polarity number, global topological charge index), three-dimensional profiles (for instance, average geometric distance degree and radius of gyration), as well as other parameters (like total positive and negative charges). All these parameters are calculated based on the Dragon software. Also, the Tanimoto coefficient (as displayed in Equation (1)) was engaged in constructing the DL model as described in our previous work [92].

(1) $$T(A,B)=\frac{A\cdot B}{{\left|A\right|}^{2}+{\left|B\right|}^{2}-A\cdot B}$$

In this equation, A displays the molecular parameters of herbal ingredients, and B represents the average molecular characteristics of 6511 molecules in DrugBank (available online: http://www.drugbank.ca) database. The higher DL index the herbal ingredient has, the larger the possibility it may have certain biological effects. Finally, molecules with large DL indexes (DL ≥ 0.18) were chosen as candidate molecules for further research. This threshold is determined by consideration of the fact that the average DL index in the DrugBank is 0.18 [92].

***Oral bioavailability.*** OB prescreening is used to determine the fraction of the oral dose of bioactive compound which reaches systemic circulation in the TCM remedy. Here, a reliable *in silico* model OBioavail 1.1 [93] which integrates the metabolism (P450 3A4) and transport (P-glycoprotein) information was employed to calculate the OB values of herbal ingredients. The optimal model was constructed based on a dataset of 805 structurally diverse drugs and drug-like compounds, which exhibits high predictability (with *R*_training_ = 0.89 and *R*_test_ = 0.85 for the internal training and external test sets, respectively, and the standard errors of estimate SEE_training_ = 0.35, SEE_test_ = 0.42, respectively). Finally, those molecules with proper oral bioavailability values (OB ≥ 30%) were selected for subsequent research.

***Caco-2 permeability.*** Due to the good correlations often existing between the human oral drugs’ adsorptions and their apparent permeability coefficients in human intestinal epithelial (Caco-2) cells [94], Caco-2 cell monolayers are widely applied as standard permeability-screening assay for prediction of the compound’s intestinal absorption and fraction of oral dose absorbed in humans [95]. In our present work, Caco-2 cell permeation values of all molecules were calculated by *in silico* model using the VolSurf approach [96]. This Caco-2 permeability model was constructed based on 100 drug compounds and displayed an optimal statistical result (*R*^2^ > 0.8) [50]. The threshold of Caco-2 permeability is set to −0.4 with regard to the fact that compound with Caco-2 ≤ −0.4 is not permeable.

### 3.3. Target Identification

In order to identify the target proteins of herbal ingredients, we developed a combinatorial approach that integrates the text-mining and chemogenomic methods. First of all, herbal ingredients were sent to the TTD (Therapeutic Target Database, available online: http://bidd.nus.edu.sg/group/ttd/) [97], DrugBank [98] and HIT (Herb Ingredient Target, available online: http://lifecenter.sgst.cn/hit/) databases for mining their corresponding targets, resulting in compound–target interactions supported by the experiments. Secondly, the target prediction was implemented for the herbal constituents using an in-house systematic model [99] which effectively integrates the chemical, genomic and pharmacological information based on Random Forest (RF) and Support Vector Machine (SVM). The optimal model with concordance of 85.83%, sensitivity of 79.62%, and specificity of 92.76% was obtained, displaying reliable predictability. Here, the compound-target interactions with SVM score ≥ 0.8 and RF score ≥ 0.7 were extracted for further research. Finally, all targets obtained from the above two steps were sent to TTD and PharmGKB (available online: http://www.pharmgkb.org) [100] databases to eliminate the noises and overlaps.

In order to increase the dependability of these predicted compound-target interactions, molecular docking was performed using GOLD5.1 software. Specific steps are as follows: firstly, the crystallographic structures of target proteins were obtained from the RCSB Protein Data Bank database (available online: http://www.pdb.org/) or were constructed using the Swiss-Model Automated Mode Serve (available online: http://swissmodel.expasy.org/) [101] if the 3D structures were not available. Secondly, before performing the docking process, crystallographic ligands were extracted and then mixed into the docking database for re-dock and polar hydrogen atoms were added. Subsequently, genetic algorithm was adopted to accomplish the ligand conformational searching for docking simulations with default parameters. Eventually, those compound-target interactions with Gold docking scores greater than 40 were selected for further research.

### 3.4. Network Analysis

In order to further interpret the complex relationships among compounds, targets, pathways and diseases, several networks were constructed. The details are as follows:

(1) Building compound–target networks which include two different nets. Firstly, drug-target network of Western medicines (D-T network) was constructed based on FDA-approved anti-inflammatory drugs and their corresponding targets which were extracted from the TTD and DrugBank databases. Secondly, in a similar way, another active ingredient-target network for *Folium Eriobotryae* (I-T network) was built based on the screened active ingredients and their corresponding validated targets.

(2) Constructing the target-pathway network (T-P network). To further identify the major signaling pathways of *Folium Eriobotryae*, we extracted 16 inflammation-related pathways from the database of KEGG (Kyoto Encyclopedia of Genes and Genomes, available online: http://www.genome.jp/kegg/). Subsequently, all targets were attempted to be mapped onto these 16 pathways, resulting in a target–pathway network.

(3) Building the target-disease network (T-D network). All target proteins were sent to databases of PharmGKB and TTD to reveal their relevant diseases, and then these targets and their corresponding diseases were used to build the target-disease network.

All these networks were constructed by the Cytoscape 2.8.1 software [102], an open software for biological data integration and validation.

## 4. Conclusions

Inflammation is a convoluted process involving hundreds of genes and connections with various complicated diseases which makes deciphering the inflammatory mechanisms more difficult. *Folium Eriobotryae*, being a potent anti-inflammatory agent, has been used in China for thousands of years. However, due to its inherent features*—i.e.*, multiple compounds and targets—its exact mechanism of action still remains unclear. In this work, for exploring the complex therapeutic mechanisms, an integrated systems pharmacology method is employed to uncover the anti-inflammatory mechanism of this probe herb. The main results are:

(1) Through ADME prescreening, 11 anti-inflammatory ingredients possessing favorable pharmacokinetic profiles, mostly flavonoids and triterpene acids, are extracted from *Folium Eriobotryae*. Meanwhile, 43 proteins targeted by these 11 compounds are identified as the potential anti-inflammatory targets of this herb.

(2) By comparing and analyzing the I-T and D-T networks, two major conclusions are obtained: (a) *Folium Eriobotryae* exhibiting anti-inflammatory effect may be through similar pharmacological mechanisms as Western medicines, reflected by the fact that six proteins (*i.e.*, COX2, ALOX5, PPARG, ICAM1, COX1 and PPARD), being the major targets, both exist in the I–T pathway of *Folium Eriobotryae* and the D–T pathway of Western medicines; (b) however, unlike mono-therapy of Western medicines, *Folium Eriobotryae* regulates dozens of anti-inflammation-related targets, and the mutual interactions and constraints among these targets may counteract or reduce the side effects that always occur with anti-inflammatory Western medicines.

(3) Through building and analyzing the T-P network, we assume that NF-κB and MAPK pathways may be major signaling pathways regulated by the ingredients of *Folium Eriobotryae* to exert anti-inflammatory effects, mainly due to the following reasons: (a) the two pathways are highly interconnected nodes in the T-P network; (b) several targets (like COX2, PPARG, TNF and RELA) involved in NF-κB and MAPK pathways are also the crucial targets of the I-T network; (c) the two pathways as the major cross-talk pathways also play critical roles in several other pathways of the T-P network.

(4) By analyzing the I-T and T-P networks, we conclude that a combinational use of herbal ingredients may offer additive or synergistic therapeutic effects for inflammation treatment. In detail, several compounds may act on the same or different targets of same signaling cascades to exhibit synergistic or additive pharmacodynamic effects.

(5) The T-D network nicely interprets why the herb* Folium Eriobotryae* can be used not only as an anti-inflammatory agent, but also for the treatment of cancer, diabetes, lung, skin, Alzheimer’s, gastrointestinal and virus diseases. By analyzing the T-D network, we can speculate that *Folium Eriobotryae* may have the therapeutic potential for cardiovascular disease, arthritis, atherosclerosis, autoimmune disease and obesity.

In summary, a systems pharmacology-based method involves systems biology approaches, combined with pharmacodynamics and pharmacokinetics evaluations, to study drugs, targets, pathways and effects. Applying systems pharmacology to herbal medicines not only provides a research platform for deciphering the molecular mechanism of herbs, but also introduces a novel way for developing new drugs from medicinal herbs, as well as for discovering combined therapeutic agents for complex human diseases. However, despite these significant developments, systems pharmacology for herbal medicines still has some limitations: (1) the pharmacodynamic interactions among herbal active ingredients have not been sufficiently taken into account, which can strongly influence the I–T network. However, large-scale experiments used to evaluate the interactions among herbal ingredients are very time-consuming and labor-intensive, or even ineffective in some circumstances due to the intricate their relationships; (2) Active ingredients can have different binding affinities with their corresponding targets, and depending on target engagement, active ingredients and their corresponding targets can be connected with a continuous approach. Nevertheless, the unified approach for quantitative target engagement of different compounds is yet to be further studied. Thus, to completely decipher the action mechanism of herbal medicines, huge amounts of work still need to be done.

## Supplementary Materials

Supplementary materials can be found at http://www.mdpi.com/1422-0067/16/02/2913/s1.
